# Diagnosis of Asthma in Primary Health Care: A Pilot Study

**DOI:** 10.1155/2014/898965

**Published:** 2014-04-10

**Authors:** Karin C. Ringsberg, Paula Bjärneman, Ronny Larsson, Elisabeth Wallström, Olle Löwhagen

**Affiliations:** ^1^Nordic School of Public Health, 402 42 Gothenburg, Sweden; ^2^Sahlgrenska Academy, University of Gothenburg, 413 90 Gothenburg, Sweden; ^3^Kungsten Health Care Centre, 414 74 Gothenburg, Sweden

## Abstract

Some patients with an asthma diagnosis have a poor controlled asthma. One explanation may be an incorrect diagnosis. * Aim.* The aim of the study was to diagnose and classify patients with non-infectious lower respiratory tract problems in primary health care using internationally applied diagnostic criteria and diagnostic tests. * Patients and Methods.* New adult patients visiting a primary health care centre due to lower airway problems were included. The diagnostic tests included FEV_1_, FVC, PEF, two questionnaires, methacholine test, and skin prick test. *Results.* The patients (*n* = 43) could be divided into four groups: asthma (28%), asthma-like disorder (44%), idiopathic cough (12%), and a nonreversible bronchial obstructive group (16%). The asthma and asthma-like groups showed similar patterns of airway symptoms and trigger factors, not significantly separated by a special questionnaire. Phlegm, heavy breathing, chest pressure/pain, cough, and wheezing were the most common symptoms. Physical exercise and scents were the dominating trigger factors. *Conclusions.* Nonobstructive asthma-like symptoms seem to be as common as bronchial asthma in primary health care. Due to the similarities in symptoms and trigger factors the study supports the hypothesis that asthma and nonobstructive asthma-like disorders are integrated in the same “asthma syndrome,” including different mechanisms, not only bronchial obstruction.

## 1. Introduction

Bronchial asthma is a common disease worldwide and good treatment could often be offered [1]. Its prevalence in the adult population in Sweden is up to 10% [2], being the highest in the north part and in younger people. The diagnosis is based on the presence of episodic breathing troubles and variable and reversible airway obstruction [1, 3]. Most cases are not difficult to diagnose and treat; however, some patients respond poorly to asthma treatment despite ongoing asthma-like symptoms. In those cases, other diagnoses must be considered [4–15]. Disorders with asthma-like symptoms mentioned in the literature are dysfunctional breathing [11, 16–18], vocal cord dysfunction [19], pseudoasthma [20], cough variant asthma [8], multiple chemical sensitivity [21], and airway sensory hyperreactivity (SHR) [22]. In GINA, the international guidelines for asthma diagnostics and treatment [1], two disorders with asthma-like symptoms are mentioned, vocal cord dysfunction (VC) [19] and hyperventilation syndrome (HVS) [7]. As the above conditions are presented under different names, it is not possible to know the prevalence. However, Marklund et al. found that every third asthmatic had been given a wrong diagnosis [14]. They also found that the older the patients, the greater the risk of an incorrect diagnosis [14]. Johansson et al. [23] estimated the prevalence of airway sensory hyperreactivity to be about 6% in the adult population. These patients often seek primary care because of asthma-like symptoms and poor quality of life [5]. Using factor analysis in a general population sample Bonde et al. [18] identified five groups of breathing-related disorders, dysfunctional breathing, odour intolerance, asthma, bronchitis, and a group with mixed symptoms. These groups should therefore be considered when investigating patients in the primary care.

In this study, an operational definition of asthma-like disorder is used, meaning symptoms that most physicians associate with asthma but where abnormal lung function cannot be demonstrated [10]. The term asthma-like disorder is henceforth used. 

*Aim*. The aim of this pilot study, preceding a larger study, was to diagnose and classify adult patients with noninfectious lower respiratory tract problems in primary health care by using international applied diagnostic criteria.

## 2. Patients and Methods

The study was performed at a primary health care centre in Sweden in a city with about half a million inhabitants. The health care centre served about 12,000 inhabitants with intermediate to high incomes. Four GPs and five nurses worked at the centre. One of the nurses and one of the physicians were specially trained in asthma and respiratory diseases.

### 2.1. Patients

Consecutive patients who visited the health care centre for the first time over a period of one year were included.

#### 2.1.1. Inclusion Criteria

Inclusion criteria included the following:

1. male or female, age ≥17,
2. respiratory symptoms from lower airways persisting for more than one month in order to exclude patients with short term symptoms,
3. not earlier diagnosed for these symptoms.

#### 2.1.2. Exclusion Criteria

Exclusion criteria included the following:

1. previously diagnosed airway disease (asthma, COPD, alveolitis, bronchiectasis, sarcoidosis, etc.),
2. ongoing airway infection,
3. other diseases that could influence the lower airways and chest mobility (heart disease, systemic immunological disease, osteoporosis, pulmonary emboli, etc.).

### 2.2. Methods

#### 2.2.1. Lung Function Test

Lung function test included the following:

1. FEV_1_ before and after inhalation of salbutamol 0.8 mg (reversibility) [1],
2. PEF morning and evening, before and after inhaled salbutamol 0.4 mg over a period of two weeks (variability) [1],
3. FVC and VC (obstruction) [1].

FEV% was calculated as FEV1/FVC or FEV1/VC (if VC was higher than FVC) after bronchodilation [24]. Lung function was measured by volume-dependent spirometry (Vitalograph) with normal reference values from Standardization of Lung Function Tests (Bull Europ. Physiopath. Resp.1983:19 suppl). PEF was measured by a Mini wright peak flow meter. All tests were performed in the same way in each patient.

As the patients included in the study were new patient they had no treatment, neither before nor during the investigation. They were prescribed therapy after completed investigation.

#### 2.2.2. Skin Prick Test

The skin prick test was performed on patients who reported a suspected allergy history. A standard panel of 10 allergen extracts (Soluprick SQ ALK Copenhagen) was used (two mites, two moulds, birch, mugwort, timothy, cat, dog, horse).

#### 2.2.3. Methacholine Test

Patients with unclear lung function data or values close to pathological limit were referred to an asthma-allergy clinic for a methacholine inhalation test. A positive test was defined as PC_20_ (provocation concentration at 20% fall in FEV_1_ ≤ 4 mg/mL) [25].

#### 2.2.4. Questionnaire

Two questionnaires were used. One general questionnaire, number 1 (see Tables 2 and 5), used for several years in the clinic included common symptoms (cough, wheezing, heavy breathing, phlegm, and chest pressure/pain) and common trigger factors (cold air, physical exercise, smoke, strong scents, emotional stress, and allergens). The patients were asked to answer yes or no. The second questionnaire, number 2, included 21 symptoms and 7 trigger factors (see Tables 3 and 6) and had earlier been designed to separate asthma from asthma-like disorders in patients referred to a special clinic for asthma and allergy [26]. The patient was asked to rate the frequency of symptoms on a five-point Likert scale: 1 = never, 2 = occasionally, 3 = once a month, 4 = once a week, and 5 = daily and the severity of the trigger factors on a five-point Likert scale: 1 = not at all, 2 = some, 3 = rather a lot, 4 = much, and 5 = very much. One question in the original version of questionnaire number 2 (strong scents) was excluded as it was also included as a trigger factor. Symptoms presented in questionnaire number 2 (Table 3) were grouped into five subsets of symptoms [26]: “upper airways nose throat”, “lower airways-chest”, “general symptoms”, “stomach”, and “sleep”.

#### 2.2.5. Diagnostic Criteria

For bronchial asthma, COPD, and chronic cough, international approved criteria were used [1, 24, 25]. For asthma-like disorders, criteria were set up on the basis of earlier studies [4, 9–11, 14, 15].

*Bronchial Asthma [1]*. Bronchial asthma included the following:

1. episodic lower airway symptoms (breathing complaints, wheezing, coughing, phlegm, chest tightness, or chest pain/pressure) [1],
2.
     
  - reversibility of FEV_1 _≥ 12% [1],
  - variability in PEF ≥ 20% [1].

*Asthma-Like Disorders [4, 9–11, 14, 15, 26]*. Asthma-like disorders include the following:

1. episodic lower airway symptoms (breathing complaints, wheezing, coughing, phlegm, chest tightness, or chest pain/pressure),
2. reversibility in FEV_1_ < 12%,
3. FEV_1_ ≥ 90% of normal predicted value,
4. variability in PEF < 20% [4, 9].

*COPD [24].* COPD included the following:

1. lower airway symptoms (breathing complaints, wheezing, coughing, phlegm, chest tightness, and chest pain/pressure),
2. reversibility in FEV_1_ < 12%,
3. variability in PEF < 20%,
4. FEV% < 70% (FEV_1_/FVC or FEV_1_/VC if VC was higher than FVC) after bronchodilation.

*Chronic Idiopathic Cough [27]*. Chronic idiopathic cough included the following:

1. dominant cough lasting ≥ 8 weeks,
2. FEV_1_ ≥ 90% of predicted normal value,
3. reversibility in FEV_1_ < 12%,
4. variability in PEF < 20%.

### 2.3. Statistics

Within-group comparisons in questionnaire number 1 (yes/no answers) were analysed using the chi-square test. The statistical analysis focused on the difference between asthma and asthma-like disorders. For differences between groups, nonparametric methods were used; for questionnaire number 2, the Wilcoxon signed-ranks test was used and for comparisons between Group I and II, Mann Whitney's test was used. *P* < 0.05 was considered to be statistically significant.

## 3. Results

**Figure 1:**
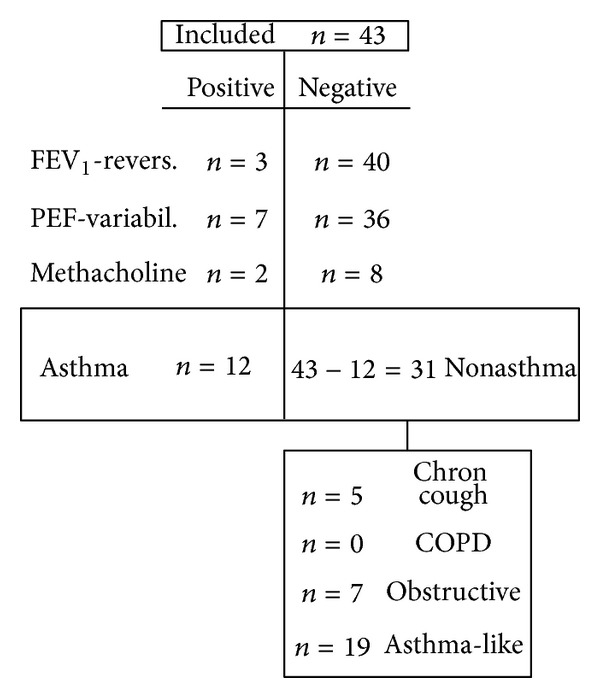
Flow chart. Classification to different groups (see Section 3) depending on FEV_1_-reversibility, PEF-variability, and methacholine test.

Of the 537 patients visiting the centre because of airway symptoms eight percent (*n* = 43) fulfilled the inclusion criteria for the study. Characteristics are shown in Table 1 and Figure 1 (flow chart).

### 3.1. Classification

Based on the diagnostic criteria above, the patients (38 women and 5 men, aged 17–83, median 54) were divided into four groups; (Group I) asthma (28%), (Group II) asthma-like (44%), (Group III) chronic idiopathic cough (12%), and (Group IV) nonreversible bronchial obstructive group (16%). No patient fulfilled the criteria for COPD. The median age was the highest in Group IV (59 years) and the lowest in Group I (44 years). The dominant sex was female in all groups. Men were only represented in Groups II and IV (Table 1).

*Group I*. Twelve patients (28%) fulfilled the diagnostic criteria for bronchial asthma. Two patients (with borderline reversibility or variability) were classified as belonging to this group on the basis of a positive methacholine tests (threshold dose ≤ 4 mg/Ml). Four patients had a positive skin prick test.

*Group II*. Nineteen patients (44%) fulfilled the criteria for an asthma-like disorder. All patients had a FEV_1_, reversibility, and PEF variability within normal range. A methacholine test was performed in six cases, and all tests were negative (threshold dose > 16 mg/mL).

*Group III*. Five patients (12%) fulfilled the criteria for a chronic idiopathic cough. The duration of the cough varied from four months to 10 years. In none of the patients were there clinical signs of a reflux, rhinitis, or sinusitis.

*Group IV*. Seven patients (16%) had a bronchial obstruction, varying in FEV_1_ from 73% to 86% predicted; however, without reversibility or variability. None of them was a smoker, none had an allergy, and none fulfilled the criteria for COPD. One patient was classified as belonging to this group on the basis of a negative methacholine test. The investigation of these obstructive patients continued and ended with the following final diagnoses: two patients with infection-induced asthma, one with asthma and lung cancer, one with probable asthma and unclear resting dyspnea, one with bronchiectasis, one with asthma, diaphragmatic hernia, and reflux, and one patient with obstruction, chronic cough, and phlegm.

### 3.2. Skin Prick Test

Eleven patients reported a positive allergy history with airway complaints. Five of these patients had a negative skin prick test and were considered nonallergic. Of the six patients with a positive test, four were found in Group I (pollen, cat, dog, and mite) and two in Group II (pollen, cat, and dog). The results of the skin prick test did not change the classification of the patients.

### 3.3. Symptoms

*Questionnaire Number 1.* The most common symptom reported in all groups was phlegm (Table 2). Cough was, by definition, the most common symptom in Group III. The second most common symptom was chest pressure/pain (Group I) and heavy breathing (Groups II and IV). Wheezing, often considered to be a criterion for asthma, was reported by 50% of Group I and by 37% of Group II. There were no significant differences between Groups I and II for any of the five most common symptoms.

*Questionnaire Number 2.* There were no significant differences in symptoms of breathing troubles (wheezing, hissing, difficulty in getting air, and difficulty in taking deep breaths) between Groups I and II (Table 3).

When symptoms in questionnaire number 2 were grouped into five subsets of symptoms [26]: “upper airways” (eye, nose, throat), “lower airways”, “general symptoms”, “stomach”, and “sleep”, no significant differences between Groups I and II were found (Table 4).

### 3.4. Trigger Factors

*Questionnaire Number 1*. The most common trigger factor in all the groups was physical exercise (walking up hills/stairs) and cold air (Table 5). There were no statistically significant differences between Groups I and II for any of these seven factors.

*Questionnaire Number 2*. The total median score of trigger factors in questionnaire number 2 (Table 6) for Group I was 1.7, for Group II 1.6, for Group III 1.1, and for Group IV 2.5. Thus, there was no difference between Groups I and II. The highest median score was seen in Group IV, “some” to “rather a lot.”

## 4. Discussion 

Most patients with respiratory complaints in Sweden are first visiting the primary health care, which has good access to practical and reliable diagnostic methods. The number of new patients in this one-year study was in line with a reported incidence of 2% of adult asthma [28] in this area. Accordingly, we believe that the results could be generalized to a larger population. Bronchial asthma and COPD are reported to be the most common chronic lower airway diseases [1, 3, 24]. In this clinical study in primary health care, the picture was different. A clear bronchial asthma was found in 28% while the majority had other respiratory complaints, asthma-like (44%), chronic idiopathic cough (12%), and nonreversible bronchial obstruction (16%). If five patients (possibly asthma) in the unclear obstructive group were added to the asthma group, the prevalence of asthma and asthma-like disorders was approximately the same. Similar findings are reported in another study in the primary care [14]. The results are also supported by a recent epidemiological study by Bonde et al. [18]. By use of factor analysis five groups of breathing related symptoms were identified, asthma, bronchitis, dysfunctional breathing, odour intolerance, and a mixed group. As the patients included in the study were new patients they had no treatment, neither before nor during the investigation. The patients were prescribed therapy after completing the investigation.

An asthma-like disorder may be an early stage of bronchial asthma or bronchial asthma in a temporary symptom-free stage with normal lung function. However, it has been shown that a disorder with asthma-like symptoms and normal lung function may persist for more than 5 years [29, 30]. New cases of COPD were not found during this study period which was somewhat unexpected. However, this may be explained by the low percentage of smokers in this area, 10%, compared to 18% in national epidemiological studies [18]. One or more patients in the obstructive group (group IV) may have incipient COPD but did not fulfil the diagnostic criteria. The non-COPD diagnosis was supported by the fact that none of the patients was a smoker. Another explanation could be obstruction due to remodelling. The extended investigation of the unclear group showed complicated and combined diseases were asthma could be suspected in 5 of the 7 cases.

In international guidelines [1, 3] two asthma-like disorders are mentioned as the most common differential diagnoses, vocal cord dysfunction [19], and hyperventilation syndrome [7, 31]. In this study there were no clear indications of these diseases. Based on the questionnaires and records, no patient reported inspiratory stridor or upper airway symptoms typical of VCD. Hyperventilation syndrome [7, 31] is a well-known asthma-like disorder since many years, but its existence in a chronic form has been contested [31, 32]. However, as it has been clearly demonstrated in acute studies hyperventilation it is still of interest [12, 13, 16, 17, 20, 31, 33–36]. By using the Nijmegen questionnaire, often used for identifying hyperventilation syndrome, Thomas et al. [16] recently found that every third woman with an asthma diagnosis had a positive score (≥23). The disorder that was identified in this way was not called hyperventilation but dysfunctional breathing. Dysfunctional breathing has also been described in other studies [7, 17, 36]. Some symptoms listed in the Nijmegen questionnaire are also included in questionnaire number 2 [26]: “difficulty in taking deep breaths,” “sensation of bloated abdomen,” “feeling of confusion,” “cold hands and feet,” and “feeling of tenseness in the body.” Thus, it is likely that the asthma-like disorder found in our study overlaps with the above described dysfunctional breathing. In 1998 the asthma-like disorder airway sensory hyperreactivity (SHR) was described by use of the capsaicin inhalation test [22]. Its prevalence has been reported to be about 6% [23], which can be compared with the prevalence of asthma of 6–10% in the same area [2]. This disorder may well be found in Group II in our study, but capsaicin inhalation test is still not available in the primary care.

International guidelines of respiratory diseases focus on asthma and COPD [1, 3]. This study points to the importance of also seeing other nonvariable and nonobstructive asthma-like disorders. The symptoms and trigger factors are very similar to asthma, which makes the differential diagnosing difficult. Bronchial obstruction, reversible and variable, is one mechanism but there might be more forming an “asthma syndrome”. This hypothesis is supported by a recent study by Bonde et al. [18]. It is important not to ignore asthma-like disorders even if they do not fit into any diagnostic criterion. The patients are often complaining of poor quality of life and that their symptoms are seen “just as psychological problems”. For the moment no medical treatment can be offered. However, alternative treatment inspired by cognitive behavioural therapy has been successful [37–39].

## 5. Conclusions

In conclusion, this study shows that asthma-like symptoms with or without reversible bronchial obstruction are equally common in patients seeking primary health care. The similarities in symptoms and trigger factors support the hypothesis that asthma and nonobstructive asthma-like disorders are integrated in the same “asthma syndrome”, which might include different mechanisms, not only bronchial obstruction.

## Figures and Tables

**Table 1:** Age, sex, lung function, and classification of patients.

Group	Age median (range)	Sex male/female	FEV_1_ % predicted mean (range)	FEV_1_ reversibility mean % (range)	PEF variability mean % (range)
(I) Asthma *N* = 12	44 (36–83)	0/12	99 (76–123)	6 (0–13)	24 (8–52)
(II) Asthma-like *N* = 19	47 (19–76)	3/16	103 (91–122)	2 (0–8)	10 (7–19)
(III) Idiopathic cough *N* = 5	50 (39–57)	0/5	103 (95–107)	3 (0–7)	11 (2–15)
(IV) Nonreversible bronchial obstructive *N* = 7	59 (17–66)	2/5	79 (73–86)	4 (0–11)	11 (5–16)

**Table 2:** The five most reported symptoms based on questionnaire no. 1. Group I–IV.

	Group I *N* = 12	Group II *N* = 19	Group III *N* = 5	Group IV *N* = 7
Phlegm	12 (100%)	18 (95%)	4 (80%)	7 (100%)
Heavy breathing	9 (75%)	16 (84%)	0 (0%)	4 (58%)
Chest pressure/pain	9 (75%)	9 (47%)	0 (0%)	2 (29%)
Cough	2 (16%)	12 (63%)	5 (100%)	3 (29%)
Wheezing	6 (50%)	7 (37%)	0 (0%)	3 (43%)

**Table 3:** Frequency of single symptoms scored on questionnaire no. 2.

Symptom	Group I *N* = 12 Median (range)	Group II *N* = 19 Median (range)	Group III *N* = 5 Median (range)	Group IV *N* = 7 Median (range)
(1) Dry eyes	1.0 (1–4)	1.0 (1–4)	1.0 (1-1)	1.0 (1-1)
(2) Dry mucus in nose	2.0 (1–5)	2.0 (1–5)	1.0 (1–4)	2.0 (1–5)
(3) Dry mucus in throat	2.0 (1–5)	2.0 (1–5)	1.0 (1–5)	3.0 (2–5)
(4) Taste of blood	1.0 (1-2)	1.0 (1–4)	1.0 (1-2)	1.0 (1-1)
(5) Difficulty in getting air	2.0 (1–5)	3.0 (1–5)	1.0 (1–4)	2.0 (1–3)
(6) Difficulty in taking deep breaths	2.0 (1–5)	2.0 (1–5)	2.0 (1–4)	2.0 (1–5)
(7) Wheezing	2.0 (1–5)	2.0(1–5)	1.0 (1-2)	1.0 (1–4)
(8) Hissing	2.0 (1–5)	2.0 (1–5)	1.0 (1-2)	1.0 (1–4)
(9) Feeling of sore airways	2.0 (1–5)	2.0 (1–5)	1.0 (1–4)	2.0 (1–5)
(10) Irritating cough	5.0 (1–5)	3.0 (2–5)	5.0 (1–5)	3.0 (2–5)
(11) Nausea	1.0 (1–3)	2.0 (1–5)	1.0 (1–4)	2.0 (1–4)
(12) Sensation of bloated abdomen	1.0 (1–4)	2.0 (1–4)	1.0 (1–5)	1.0 (1-2)
(13) Waking up due to nasal congestion	2.0 (1–5)	2.0 (1–5)	2.0 (1–5)	2.0 (1–5)
(14) Abnormal tiredness, weakness after psychological stress	2.0 (1–5)	2.0 (1–5)	1.0 (1–4)	2.0 (1–4)
(15) A sore throat	2.0 (1–5)	2.0 (1–5)	1.0 (1–4)	2.0 (1–5)
(16) Headaches	2.0 (1–4)	2.0 (1–5)	3.0 (2–4)	2.0 (1–3)
(17) Feeling of confusion	1.0 (1–4)	1.0 (1–4)	1.0 (1-1)	2.0 (1–3)
(18) Cold hands and feet	2.0 (1–5)	2.5 (1–5)	2.0 (1–5)	2.0 (1–4)
(19) Feeling of tenseness in the body	2.0 (1–5)	3.0 (1–5)	2.0 (1–3)	2.5 (1–3)
(20) Difficulty in concentrating	2.0 (1–4)	2.5 (1–5)	2.0 (1–4)	2.0 (1–5)

**Table 4:** Subsets of symptoms scored in questionnaire no. 2.

Subsets of symptoms (questions number)	Group I *N* = 12 Median (range)	Group II *N* = 19 Median (range)	Group III *N* = 5 Median (range)	Group IV *N* = 7 Median (range)
Upper airways, eyes, nose, throat (1, 2, 3, 4)	2.0 (1–5)	1.0 (1–5)	1.0 (1–5)	1.0 (1–5)
Lower airways (5, 6, 7, 8, 9, 10)	2.0 (1–5)	2.0 (1–5)	1.0 (1–5)	2.0 (1–5)
General symptoms (16, 17, 18, 19, 20)	2.0 (1–5)	2.0 (1–5)	2.0 (1–5)	2.0 (1–5)
Stomach (11, 12)	1.0 (1–4)	2.0 (1–5)	1.0 (1–5)	1.0 (1–4)
Sleep (13, 14)	2.0 (1–5)	2.0 (1–5)	2.0 (1–5)	2.0 (1–5)

**Table 5:** Most reported symptom-inducing trigger factors based on questionnaire no. 1.

Trigger factor	Group I *N* = 12 *n* (%)	Group II *N* = 19 *n* (%)	Group III *N* = 5 *n* (%)	Group IV *N* = 7 *n* (%)
Walking up hills/stairs	10 (83)	10 (53)	2 (40)	6 (86)
Cold air	7 (58)	8 (42)	3 (60)	4 (57)
Mental stress	3 (25)	4 (21)	1 (20)	4 (57)
Tobacco smoke	3 (25)	7 (37)	1 (20)	4 (57)
Flowers	3 (25)	4 (21)	0 (0)	5 (71)
Perfume	3 (25)	0 (0)	0 (0)	5 (71)
Exhaust gases	3 (25)	3 (16)	0 (0)	2 (29)

**Table 6:** Severity of symptom induced trigger factors based on questionnaire no. 2.

Trigger factor	Group I *N* = 12 Median (range)	Group II *N* = 19 Median (range)	Group III *N* = 5 Median (range)	Group IV *N* = 7 Median (range)
(1) Warm weather	1.0 (1–5)	1.0 (1–4)	1.0 (1-2)	1.5 (1–4)
(2) Conflicting situations	1.5 (1–4)	1.0 (1–4)	1.0 (1–3)	1.5 (1–4)
(3) Strong scents	2.5 (1–5)	2.0 (1–4)	1.0 (1-1)	3.0 (2–5)
(4) Exhaust gases	2.0 (1–5)	2.0 (1–4)	1.0 (1–3)	3.0 (2–5)
(5) Stuffy air	2.0 (1–4)	2.0 (1–3)	1.0 (1-1)	3.0 (1–5)
(6) Smell of tobacco	2.0 (1–5)	2.0 (1–5)	2.0 (1–4)	3.5 (2–5)
(7) Dust from detergent	1.0 (1–4)	1.0 (1–3)	1.0 (1-1)	2.0 (1–5)

*P* = ≤0.05.
